# Preoperative visualization of congenital lung abnormalities: hybridizing artificial intelligence and virtual reality

**DOI:** 10.1093/ejcts/ezad014

**Published:** 2023-01-16

**Authors:** Wouter Bakhuis, Casper M Kersten, Amir H Sadeghi, Quinten J Mank, René M H Wijnen, Pierluigi Ciet, Ad J J C Bogers, J Marco Schnater, Edris A F Mahtab

**Affiliations:** Department of Cardiothoracic Surgery, Thoraxcenter, Erasmus MC, Rotterdam, Netherlands; Department of Pediatric Surgery, Erasmus MC—Sophia Children’s Hospital, Rotterdam, Netherlands; Department of Cardiothoracic Surgery, Thoraxcenter, Erasmus MC, Rotterdam, Netherlands; Department of Cardiothoracic Surgery, Thoraxcenter, Erasmus MC, Rotterdam, Netherlands; Technical Medicine, Delft University of Technology, Delft, Netherlands; Department of Pediatric Surgery, Erasmus MC—Sophia Children’s Hospital, Rotterdam, Netherlands; Department of Radiology and Nuclear Medicine, Erasmus MC Sophia Children’s Hospital, Rotterdam, Netherlands; Department of Cardiothoracic Surgery, Thoraxcenter, Erasmus MC, Rotterdam, Netherlands; Department of Pediatric Surgery, Erasmus MC—Sophia Children’s Hospital, Rotterdam, Netherlands; Department of Cardiothoracic Surgery, Thoraxcenter, Erasmus MC, Rotterdam, Netherlands

**Keywords:** Virtual reality, Paediatric lung surgery, Congenital lung abnormalities, Congenital pulmonary airway malformations, Segmentectomy

## Abstract

**OBJECTIVES:**

When surgical resection is indicated for a congenital lung abnormality (CLA), lobectomy is often preferred over segmentectomy, mostly because the latter is associated with more residual disease. Presumably, this occurs in children because sublobar surgery often does not adhere to anatomical borders (wedge resection instead of segmentectomy), thus increasing the risk of residual disease. This study investigated the feasibility of identifying eligible cases for anatomical segmentectomy by combining virtual reality (VR) and artificial intelligence (AI).

**METHODS:**

Semi-automated segmentation of bronchovascular structures and lesions were visualized with VR and AI technology. Two specialists independently evaluated via a questionnaire the informative value of regular computed tomography versus three-dimensional (3D) VR images.

**RESULTS:**

Five asymptomatic, non-operated cases were selected. Bronchovascular segmentation, volume calculation and image visualization in the VR environment were successful in all cases. Based on the computed tomography images, assignment of the CLA lesion to specific lung segments matched between the consulted specialists in only 1 out of the cases. Based on the three 3D VR images, however, the localization matched in 3 of the 5 cases. If the patients would have been operated, adding the 3D VR tool to the preoperative workup would have resulted in changing the surgical strategy (i.e. lobectomy versus segmentectomy) in 4 cases.

**CONCLUSIONS:**

This study demonstrated the technical feasibility of a hybridized AI–VR visualization of segment-level lung anatomy in patients with CLA. Further exploration of the value of 3D VR in identifying eligible cases for anatomical segmentectomy is therefore warranted.

**Figure:**
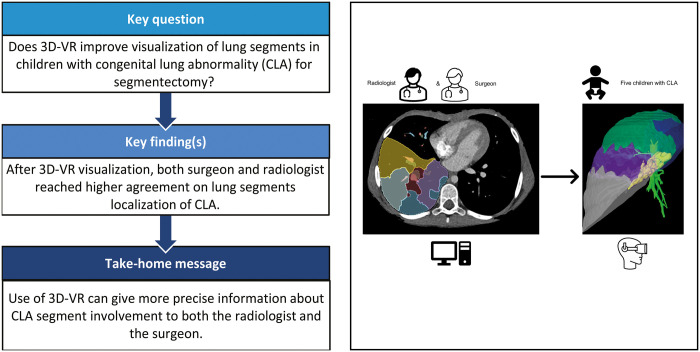

## INTRODUCTION

Congenital lung abnormalities (CLA) are developmental pulmonary lesions, which comprise, among others, congenital pulmonary airway malformation (CPAM), bronchopulmonary sequestration (BPS), bronchogenic cyst and congenital emphysema. CPAM is the most common of these, though hybrid lesions—showing characteristics of multiple CLA entities—are common as well [1, 2].

The incidence of CLA has increased in recent years to ∼4 in 10,000 births, presumably because the performance of antenatal ultrasound-guided diagnostics has improved [3]. If an infant remains asymptomatic after birth, which is true for the majority of cases, the choice between surgical intervention and conservative wait-and-see management is not standardized and highly depends on local clinical guidelines [4–8]. However, if symptoms occur in the first years of life (i.e. neonatal respiratory distress, persistent cough or recurrent lung infections), surgical resection is indicated [8]. Lobectomy is then the treatment of first choice, either through thoracotomy or minimally invasive video-assisted thoracoscopic surgery [5].

Recent studies on adult pulmonary oncology have proven that lung-sparing segmentectomy is a feasible and effective alternative to lobectomy in specific cases [9, 10]. Interestingly, only a few cases of sublobar resection through segmentectomy have been described in the CLA population [11, 12]. Furthermore, residual disease following sublobar resection in CLA cases has been reported in up to 15% of cases, even though CLA is considered a benign lesion [5]. Suboptimal knowledge of anatomy in infants and preoperative localization of the lesions based on current imaging techniques could play a role herein [13].

Accurate image-guided preoperative planning is required to identify suitable patients and to optimally prepare for segmentectomy. Currently, preoperative planning in paediatric pulmonary surgery is most commonly aided by contrast-enhanced computed tomography imaging of the chest [chest contrast-enhanced computed tomography (CECT)]. Recent advances in imaging have enabled three-dimensional (3D) reconstruction and virtual reality (VR) visualization of patient-specific anatomy [14–18]. Moreover, emerging artificial intelligence (AI) algorithms allow automated segmentation of anatomic structures and consequently facilitate the fast analysis of computed tomography (CT) scans, without the need for extensive manual segmentation [19].

We have recently combined VR and AI technology to study the feasibility and clinical efficacy of a VR-AI-based hybrid platform (PulmoVR) in the preoperative planning of pulmonary segmentectomy in adults [18]. We hypothesize that PulmoVR could potentially serve as a preoperative planning tool for identifying children with CLA who qualify for an anatomical segmentectomy, thereby conserving lung tissue [20, 21].

The aim of this study is to prove the feasibility of using a hybrid approach based on AI, (semi-)automatic segmentation and VR to realize immersive 3D representations of patient-specific lung anatomy in children with CLA. Moreover, we investigated if VR imaging is of any added value for the preoperative planning of anatomical segmentectomy in these children.

## MATERIALS AND METHODS

### Ethics statement

This study was approved by the local medical ethics committee of the Erasmus University Medical Center, Rotterdam, Netherlands (MEC-2020-0891). Verbal informed consent was obtained from the parents or guardians of the participating children.

### Patient selection

We selected for this feasibility study children who prenatally had been diagnosed with CLA during routine ultrasound imaging. The selection was through a spirometry-controlled, chest CECT. Diagnosis was confirmed on radiological imaging and no tissue diagnosis was performed, as no patients underwent surgery or biopsy yet.

### Computed tomography scan segmentation

After obtaining a chest CT, the anonymized images were uploaded in digital imaging and communications in medicine format to the Medical Image Communication Platform EVOCS (Fysicon, Oss, Netherlands). Using LungQ software (Thirona, Nijmegen, Netherlands), an AI-based segmentation of bronchial, venous and arterial segmental branches was executed, whereby the segmental borders were determined for the calculation of segmental volumes. The anatomical 3D segmentations were visually checked for accuracy by a trained analyst within Thirona's ISO 13485-certified image analysis service. The process of data exchange, automatic segmentation and validation took <48 h. Simultaneously, semi-automatic segmentation of the CLA lesions was performed by physicians and clinical technologists by using the open-source 3D-Slicer segmentation software [22]. The segmentation was then validated by a second reader, according to a previously published protocol [23]. Segmentation of these CLA lesions required <1 h time.

### Three-dimensional model and virtual reality rendering and visualization

VR visualization was achieved by using our own VR workstation and software (PulmoVR), which has been jointly developed at our Department of Cardiothoracic Surgery in collaboration with MedicalVR (Amsterdam, Netherlands), Thirona (Nijmegen, Netherlands) and Fysicon (Oss, Netherlands). Furthermore, interactive 3D reconstructions for visualization on a monitor were made by using ‘Pulmo3D’ software, jointly developed by the department of Cardiothoracic Surgery, MedicalVR and Thirona. The figures are 2D computer screenshots of the 3D-VR environment in PulmoVR. Pulmo3D software was used to visualize an interactive 3D model.

### Study design

Our main objective was to study the technical feasibility and theoretical clinical usefulness of 3D-VR for preoperative planning of CLA surgery. Technical feasibility was defined as: (i) successful automatic (AI-based) segmentation of (abnormal) bronchovascular anatomy, based on chest CECT images of children with known CLA; (ii) successful automatic (AI-based) delineation of lung segments based on chest CECT images of children with known CLA; (iii) successful semi-automatic segmentation and visualization of CLA-specific lesions; and (iv) successful rendering of segmentation files and chest CECT images in VR platform PulmoVR and Pulmo3D software.

If images had been successfully rendered, a paediatric thoracic surgeon and a paediatric thoracic radiologist evaluated these images, with 15 and 10 years of experience in CLA, respectively. First, they assessed the 2D-CT images (normal CT scan, using the standard CT viewer, thus without segmentation of structures of interest) of the 5 cases in random order and completed a short questionnaire in which they could comment on the image quality and lesion localization. Then, they assigned the lesion to 1 or more lobes and segments **(**Supplementary Material, Questionnaire A**).** After at least 1 week, both specialists viewed the 3D-VR images (including segmentations of interest) of the 5 cases in random order and filled in the same survey on image quality and lesion localization.

### Data analysis

The results of the questionnaires were compared both between observers per imaging modality, and for each observer separately between the different images. All data were analysed using Microsoft Office Excel (Microsoft, Redmond, Washington, USA). The kappa statistic was used to calculate interobserver agreement (surgeon versus radiologist) and calculated using statistical package IBM SPSS Statistics software (SPSS) [24]. Kappa statistic can be interpreted as a range where 0 is agreement is equal to chance and 1 is full agreement.

## RESULTS

We included 5 patients. An overview of the diagnosis and CLA localization based on chest CT is shown in Table 1. The median age at chest CT was 8 years (P25–75: 9.5, 15.5). Automatic (AI-based) and automatic segmentation of bronchovascular anatomy, segmental delineation, semi-automatic segmentation of CLA-specific lesions and segmental volume calculation were performed successfully (Supplementary Material, Fig. SA) in all patients. After segmentation, all chest CT images and segmentation files were successfully rendered in PulmoVR and Pulmo3D software. In addition, all individual pulmonary segment volumes were visualized.

**Table 1:** Overview of included patients and location of congenital lung abnormality based on computed tomography and virtual reality

Patient #	Sex	Age at CT scan (years)	Diagnosis	Location on CT scan
1	Female	8	CPAM	RLL
2	Male	8	CPAM	LUL
3	Female	12	BPS	RLL
4	Female	5	CPAM	RLL
5	Male	8	BPS	LLL

CPAM: Congenital pulmonary airway malformation; CT: computed tomography; BPS: bronchopulmonary sequestration; LLL: left lower lobe; LUL: left upper lobe; RLL: right lower lobe.

**Figure 1:**
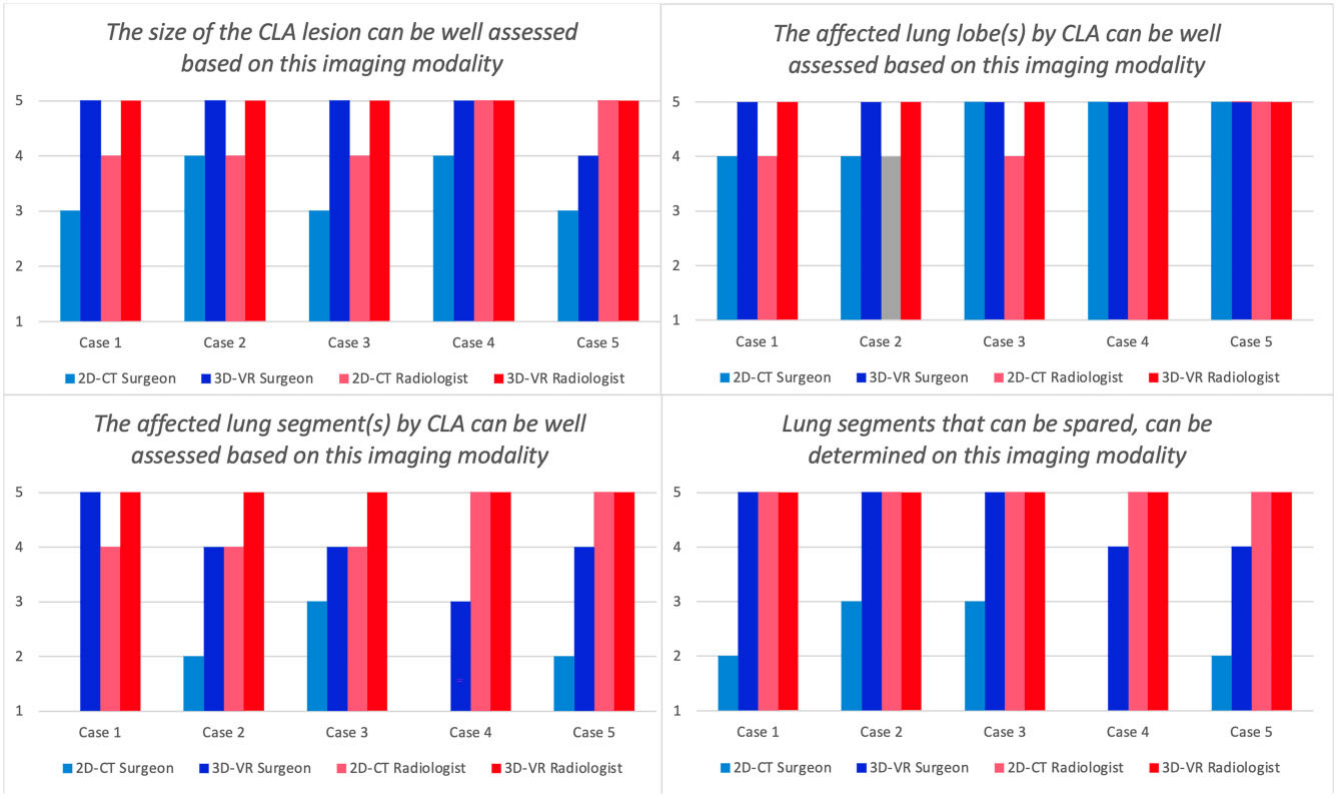
Assessment of CLA lesion size, affect lung lobes and lung segments and determination of lung segment sparing based on both imaging modalities. In the questionnaire, the paediatric thoracic surgeon and the radiologist scored the 5 cases from 1 (strongly disagree) to 5 (strongly agree). 2D-CT: two-dimensional computed tomography; 3D-VR: three-dimensional virtual reality; CLA: congenital lung abnormality

3D-VR overall tended to perform better than 2D-CT regarding image quality and usefulness for defining the lesion size (Fig. 1). Both consulted medical specialists in all cases assigned the lesion to the same lung lobe, irrespective of imaging modality (Table 2). Localization of the lesion in a specific lung segment proved to be dependent on the imaging modality, however, as both consulted specialists in almost all cases (5/5 for the surgeon and 4/5 for the radiologist) scored additional or different affected segments after viewing the 3D-VR images (Table 2). This included in 2 cases the identification of an additional segment X, as part of an anatomical variant, which was not described on 2D-CT. Furthermore, segment localization based on 2D-CT differed between the surgeon and radiologist in 4 out of 5 cases (kappa = 0.17), while the localization matched in 3 out of 5 cases after 3D-VR analysis (kappa = 0.55). Regarding a theoretically preferred surgical strategy, the paediatric thoracic surgeon in 4 out of 5 cases changed the preferred strategy based on the 2D-CT images after viewing the 3D-VR images (Table 3). In 3 cases, this concerned a switch from lobectomy resection to segmentectomy. In 1 case, this concerned a switch from segmentectomy lobectomy.

**Table 2:** Affected lung lobes and segments based on different imaging modalities

	In which lung lobe(s) do you think the CLA lesion is located?				In which lung segment(s) do you think the CLA lesion is located?			
	Paediatric thoracic surgeon		Paediatric thoracic Radiologist		Paediatric thoracic surgeon		Paediatric thoracic Radiologist	
	2D-CT	3D-VR	2D-CT	3D-VR	2D-CT	3D-VR	2D-CT	3D-VR
Case 1	RLL	RLL	RLL	RLL	7, 8	7, 8, 10	7, 8	7, 8
Case 2	LUL	LUL	LUL	LUL	4, 5	1, 2, 3, 4, 5	1, 2, 4	1, 2, 3, 4, 5
Case 3	RLL	RLL	RLL	RLL	7, 8	6, 7	7	6, 7
Case 4	RLL	RLL	RLL	RLL	6, 7, 9, 10	6, 7, 10, *X*	7, 10	6, 7, 10, *X*
Case 5	LLL	LLL	LLL	LLL	9, 10	6, 10, *X*	7, 10	7, 10, *X*

In the questionnaire, the paediatric thoracic surgeon and the radiologist localized the CLA lesions in 5 patients per lung lobe and per lung segment, based on 2D-CT and 3D-VR.

2D-CT: two-dimensional computed tomography; 3D-VR: three-dimensional virtual reality; CLA: congenital lung abnormality; LLL: left lower lobe; LUL: left upper lobe; RLL: right lower lobe.

**Table 3:** Preoperative surgical plan per image modality

	Would you perform segmentectomy?	
	If yes: of which segments?	
	If no: lobectomy?	
	2D-CT	3D-VR
Case 1	No: RLL lobectomy	Yes: S7 + S8 + S9 + S10 (basal) segmentectomy
Case 2	Yes: S4 + S5 segmentectomy	No: LUL lobectomy
Case 3	No: RLL lobectomy	Yes: S6 + S7 segmentectomy
Case 4	No: RLL lobectomy	No: RLL lobectomy
Case 5	No: LLL lobectomy	Yes: S6 + S10 + SX segmentectomy

The paediatric thoracic surgeon’s surgical plan, based on the 2D-CT or 3D-VR imaging.

2D-CT: two-dimensional computed tomography; 3D-VR: three-dimensional virtual reality; LLL: left lower lobe; LUL: left upper lobe; RLL: right lower lobe; S: segment.

**Figure 2:**
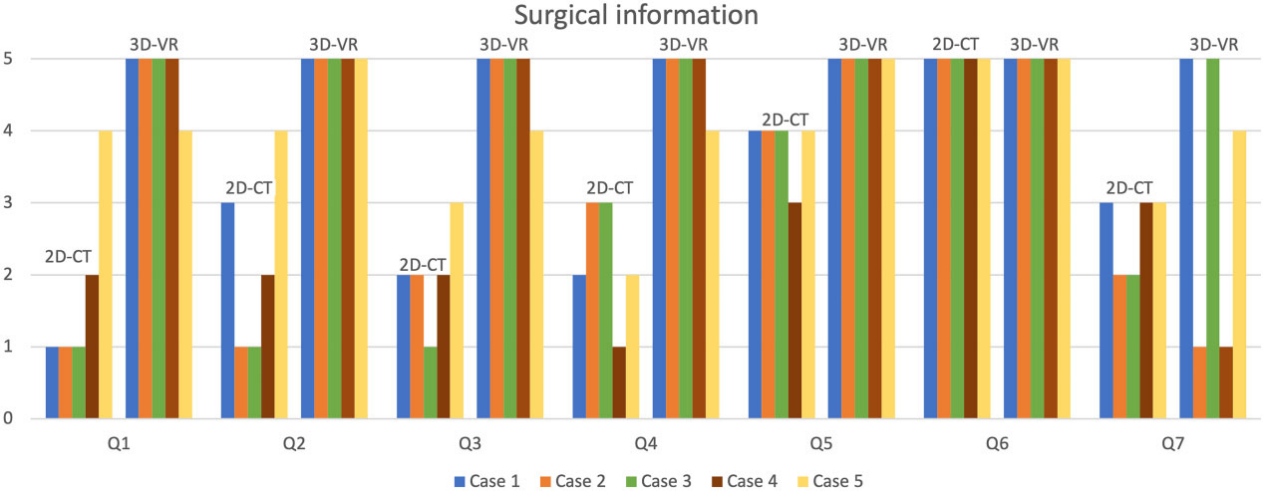
Surgical information among different imaging modalities. Surgical information about the CLA lesions was gathered from the questionnaire filled in by the paediatric thoracic surgeon. Scored from 1 (strongly disagree) to 5 (strongly agree). Q1: the pulmonary artery branches that need to be ligated can be determined on this imaging modality; Q2: the pulmonary vein branches that need to be ligated can be determined on this imaging modality; Q3: the bronchial branches that need to be ligated can be determined on this imaging modality; Q4: surgical strategy [(multi)segmentectomy or lobectomy] can be decided upon based on this imaging modality; Q5: this modality prepares me for surgery; Q6: I am likely to consult this scan in the preoperative planning; and Q7: I am likely to consult this scan during surgery. 2D-CT: two-dimensional computed tomography; 3D-VR: three-dimensional virtual reality; CLA: congenital lung abnormality; LLL: left lower lobe; LUL: left upper lobe; Q: question; RLL: right lower lobe.

The 3D-VR images provided better insight in the arterial, venous and bronchial branches that needed to be ligated than did the 2D-CT images (Fig. 2). The surgeon valued determining the preoperative strategy based on the 3D-VR images higher than that on the basis of the 2D-CT images alone. Based on AI-based segment delineation and semi-automatic CLA lesion segmentation, on average 34% of the lesion-encompassed lobe was lesion free (range 0–64%), corresponding with an average non-affected lung volume of 257 ml (Supplementary Material, Table SA). After evaluation of both the 2D-CT and 3D-VR images, the paediatric thoracic surgeon preferred to perform a segmentectomy in patients 1, 3 and 5, in whom nearly half of the volume of the affected lung lobe (range 27–64%) could potentially be spared if a segmentectomy would be performed rather than a lobectomy.

### Patient 1

This scan showed a CPAM and aberrant venous drainage of the right lower lobe (RLL) to the inferior vena cava, resembling a partial anomalous pulmonary venous return as seen in scimitar syndrome (Video 1 and Supplementary Material, Fig. SB). Based on 2D-CT images, segments 7 and 8 were affected by the CPAM. The 3D-VR images confirmed that the CPAM and its aberrant vein were located in segments 7 and 8, but also visualized some small cysts in segment 10. If surgery was indicated, basal segmentectomy could be performed instead of lobectomy, sparing segment 6, hereby conserving 26% lung tissue of the RLL (Supplementary Material, Table SB).

### Patient 2

3D-VR imaging showed that the CPAM was located in all lung segments of the left upper lobe, instead of only segments 4 and 5 (surgeon) and segments 1, 2 and 4 involvement (radiologist) as concluded from the 2D-CT images alone (Video 2 and Supplementary Material, Fig. SC). Both noticed involvement of additional segments on 3D-VR in contrast to 2D-CT images. For the surgeon, this was reason to switch from lingulectomy (resection of segments 4 and 5) to lobectomy.

### Patient 3

**Figure 3:**
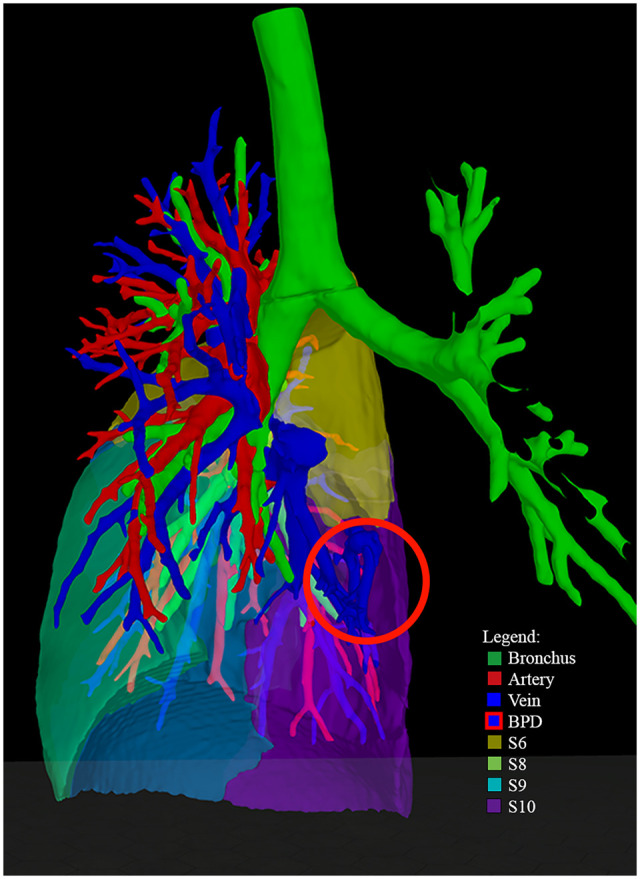
Right lower lobe segmentation: BPS of patient 3. Frontal Pulmo3D view after disabling S7, with BPS encircled, showing that the BPS is located in the right lower lobe. Segment 6 is affected as well, but arterial supply of BPS is not visualized. BPS: bronchopulmonary sequestration.

The CT scan showed BPS in the RLL, with arterial supply from the descending thoracic aorta (Fig. 3 and Video 3). 3D-VR visualization revealed that the BPS was only located in segments 6 and 7. The paediatric thoracic surgeon switched the preferred surgical strategy from lobectomy (after viewing only the 2D images) to segmentectomy (based on the 3D-VR images). This switch could potentially spare 64% of the lung volume (Supplementary Material, Table SB).

### Patient 4

**Figure 4:**
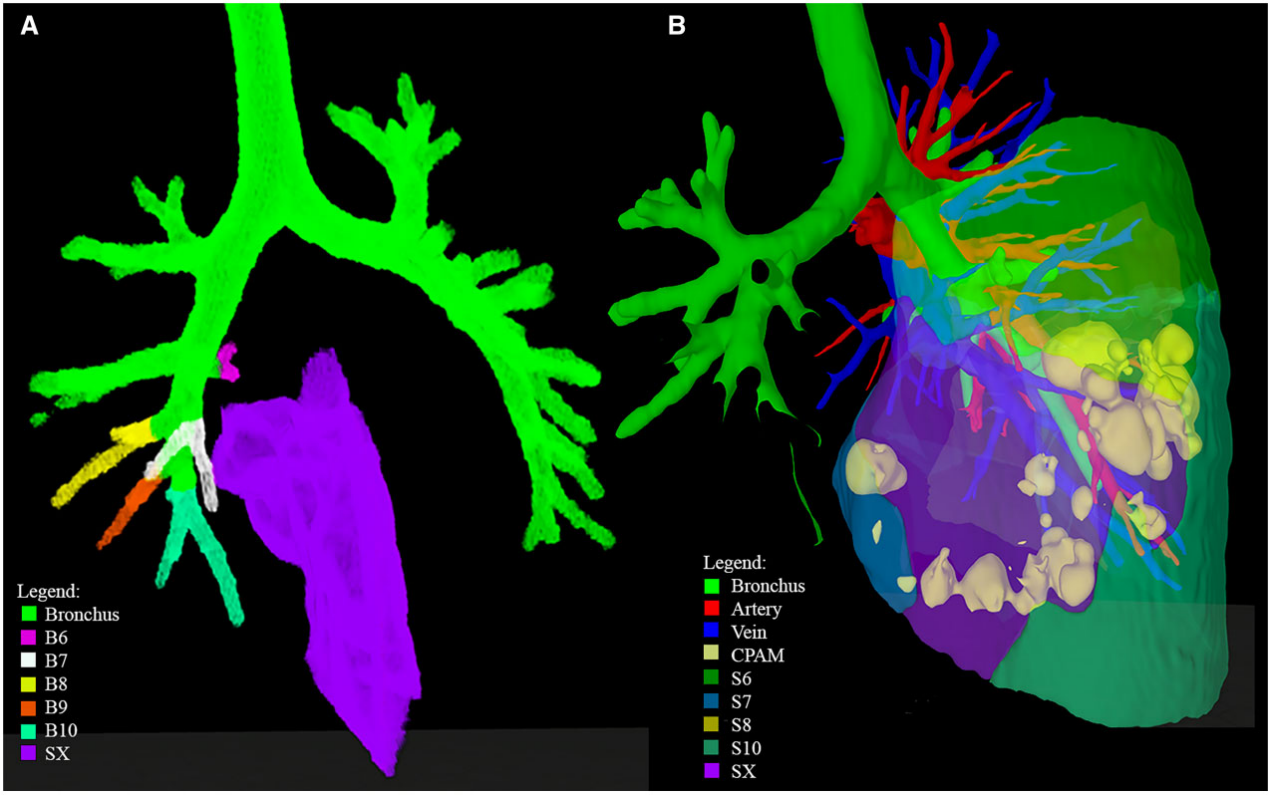
Right lower lobe segmentation: CPAM in extra lung segment of patient 4. (**A**) Frontal PulmoVR view, showing all bronchial branches of right lower lobe. Five bronchial branches were visualized (B6–B10), although 6 segments were reconstructed (SX). (**B**) Dorsal Pulmo3D view, showing CPAM involvement in all 4 visible segments (S6, S7, S10 and SX). CPAM: congenital pulmonary airway malformation; S: segment.

The CT scan confirmed a CPAM in the RLL. The 3D-VR images with visualization of the segmental delineation revealed an additional lung segment ‘X’, which brought the number of segments in the RLL from 5 to 6 (Video 4 and Fig. 4). This higher number could be ascribed to additional arterial and venous branches, since an additional bronchial branch was not observed (Fig. 4A). The CPAM was located in several segments (6, 7, 10 and X) of the RLL (Fig. 4B). In this case, the paediatric thoracic surgeon preferred a lobectomy after assessing both the 2D-CT and the 3D-VR images.

### Patient 5

**Figure 5:**
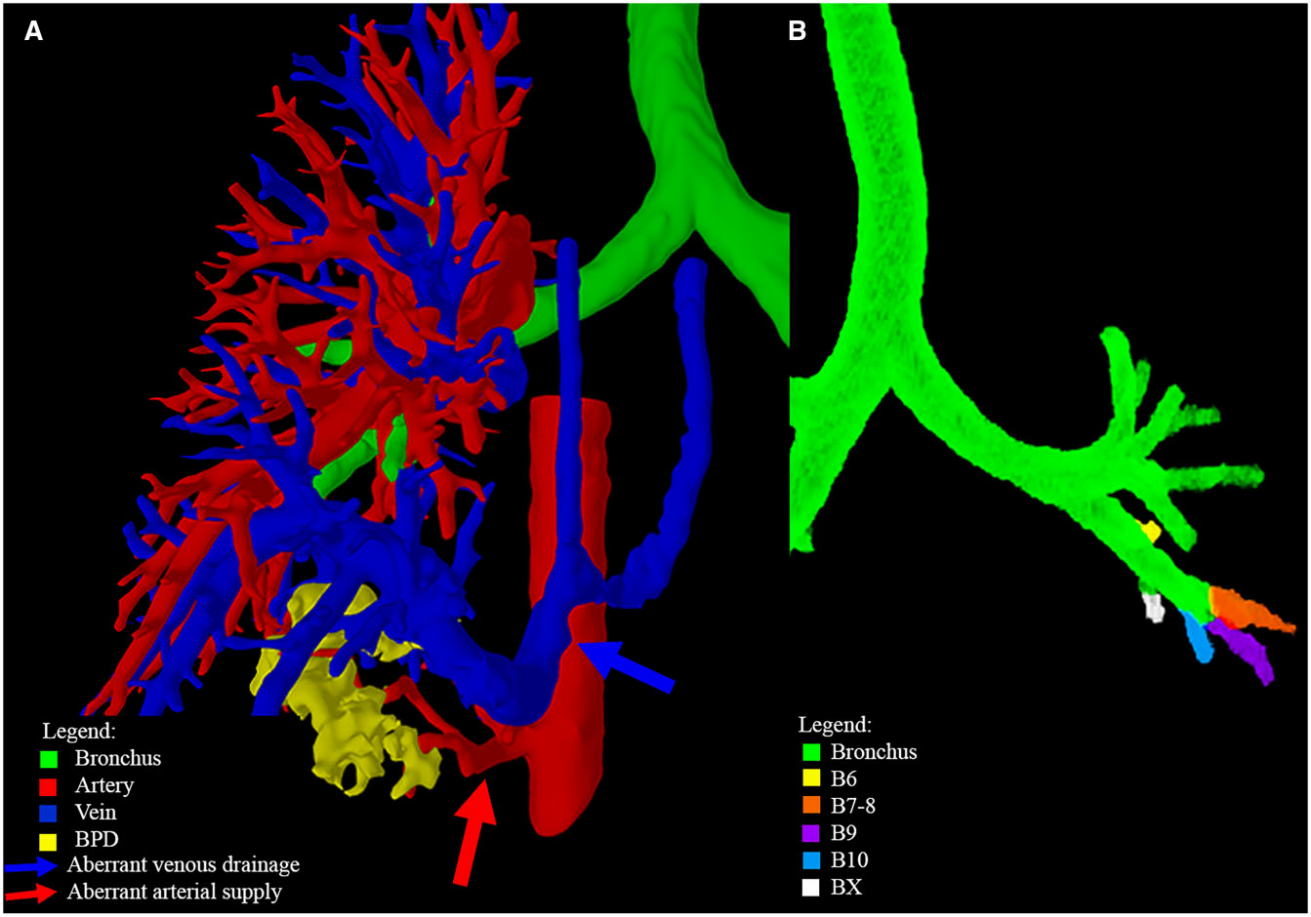
Left lower lobe segmentation: BPS of patient 5. (**A**) Dorsal Pulmo3D view showing the relation of the BPS with the aberrant arterial supply and venous drainage. (**B**) Frontal PulmoVR view of bronchial tree, with all bronchial branches of left lower lobe coloured. Additional bronchial branch is visible in white. 3D: three-dimensional; BPS: bronchopulmonary sequestration.

CT images showed a BPS in the left lower lobe (LLL) (Video 5). Aberrant venous drainage of a large pulmonary vein into the hemiazygos vein was observed. Arterial supply originated from the descending aorta (Fig. 5). Again, 3D-VR imaging discovered an additional lung segment ‘X’, also affected by the BPS. In this case, the LLL consisted of 5 bronchial branches rather than the usual 4 (Fig. 5B). None of the specialists had noted this additional segment on the 2D-CT images. Upon analysis of the 3D-VR images models, both specialists confirmed that segments 10 and X were affected by the BPS. The surgeon thought that segment 6 was affected as well, and the radiologist thought that segment 7 was affected. The researchers (Wouter Bakhuis and Casper M. Kersten) concluded that segments 9, 10 and *X* were affected by the BPS. After viewing the 2D-CT images, the surgeon preferred a lobectomy but switched to a segmentectomy after 3D-VR visualization. Trisegmentectomy of segments 9, 10 and *X* instead of an LLL lobectomy could, hypothetically, spare 52% of the volume of the LLL (Supplementary Material, Table SB).

## DISCUSSION

CLA visualization in PulmoVR appeared to be helpful for selecting CLA patients for lung-sparing segmentectomy instead of conventional lobectomy. Based on the 3D-VR assessment, in addition to the regular 2D-CT images, the paediatric thoracic surgeon changed the preferred surgical approach in 80% (4/5 patients) of the cases. In 3 cases, an initially preferred lobectomy strategy was altered to a segmentectomy; in 1 case, the opposite was true because additional involved segments were identified on the 3D-VR images. Although the surgeon and the radiologist did agree on CLA localization only in 60% cases (3/5 patients) after 3D-VR visualization (kappa = 0.55), the extent of agreement increased 40% than after sole 2D-CT visualization, when was 20% (1/5 patients) (kappa = 0.17). AI-based delineation of lung segments and intersegmental borders identified an additional lung segment with normal pulmonary vascularization in 2 out of 5 patients, both of which were affected by CLA. The current literature does not contain reports on additional lung segments—in either paediatric or adult cases.

3D-VR visualization is based on the same information as the 2D-CT scan but helps the viewer with the mental process of converting 2D slices into a 3D model and provides intuitive options to manipulate the model using the VR controllers. In addition, via visualization of AI-based segmental borders in 3D-VR, the caregiver is supported in determining lesion segment location. Even though segmental borders can be estimated based on bronchovasculature on 2D-CT, this remains very difficult, as is shown in this study by our experienced paediatric thoracic radiologist, who changed 80% of number of affected segments by CLA after 3D-VR visualization.

We stress that these preliminary results remain hypothetical, since no paediatric VR-guided lung resection was performed (yet). Nevertheless, we recently published the results of 50 VR-guided segmentectomies in adults, finding confirmed radical tumour resection in 98% of cases, based on pathological examination [25]. If these results can be reproduced remains to be investigated, due to different patient groups (children versus adults) and different lesion types (CLA versus solid tumour). The findings of our study should therefore be cautiously interpreted and replicated in a larger prospective study. Currently, we aim to validate the AI-based visualization in a cohort of CLA patients who undergo surgery. Direct comparison between imaging and pathology findings will enable assessment of agreement in the definition of the segmental borders and CLA size between the 2D-CT, 3D-VR and the intraoperative view.

If pulmonary resection is indicated for a patient with CLA, the optimal procedure should be curative whilst preserving as much healthy lung tissue as possible to improve the long-term lung function. Following this rule, segmentectomy could be preferred over a lobectomy in selected CLA cases. Recent literature in adults has proven the feasibility of lung-sparing segmentectomy as an alternative to lobectomy in oncologic cases [9, 10]. Although segmentectomy for CLA has been described, an often posed counter-argument is the risk of residual disease, which can be as high as 15% [5, 26–28]. Although the increased technical difficulty of segmentectomy plays likely a role in this high recurrence rate, another possible cause could be related to non-anatomical resections of CLA [26, 29]. In addition, as we learnt from the present study, preoperative imaging with regular 2D- CT images could also lead to the inaccurate localization of CLA lesions and therefore contribute to incomplete resections.

There is momentarily no consensus on the optimal management of asymptomatic patients [6, 7]. Surgical resection in asymptomatic cases is preferably planned before 10 months of age, as this is the average age of symptom development [5, 30]. In this study, we included only children (older than 5 years of age) for whom spirometry-controlled CECT scans were available, as these scans lead to higher-quality images, essential to successfully achieve semi-automated segmentation and VR visualization. We plan to further optimize the (semi-)automated segmentation process for non-spirometry-controlled CT images. Simultaneously, new techniques, including photon-counting detector computed tomography, will lead to improved resolution [31].

Although several studies have investigated the use of 3D-VR imaging of pulmonary lesion in the adult population, to the best of our knowledge only 1 other study has implemented these techniques for the visualization of paediatric CLA cases [32]. Similarly to our study, the authors reported preoperative VR visualization of specific anatomical structures. Nevertheless, the authors focused the image analysis on critical structures that were expected to be encountered during surgery and thus did not analyse specific segmental involvement of the lesion and potential lung-sparing surgical options.

As segmentation of CLA was executed semi-automatically in this study, this process was quite time-consuming. We therefore strive to create algorithms aimed at automating the segmentation process of CLAs. Due to the heterogeneous morphology among the different subtypes of CLA, it would be necessary to create a specific algorithm for each type of CLA. One of our long-term goals is to convert the VR visualization into augmented reality that can be projected over the surgical field during thoracoscopic surgery. In addition, we wish to implement VR-imaging tools for education and training of paediatric pulmonologists, radiologists and surgeons, to increase the awareness and expertise of these abnormalities and their associated anatomical variations. The hardware and software can be purchased commercially and is worldwide available, but the image analysis methods as described in this study can only be used for research purposes yet. The VR setup requires an initial hardware investment of approximately €2000–4000 for off-the-shelf computers and VR headsets. In addition, the software (MedicalVR, Amsterdam, Netherlands) can be licenced for a research/education-only purpose. A clinical application licence will most likely become available within the next year in European countries, after CE certification. The rendering of CT scans for a specific procedure takes ∼30 s and requires that the surgeon exports the CT scan data from their local picture archiving and communication system. Additional segmentations or analyses can be performed according to surgeon’s preference and the complexity of the cases and are therefore suitable for all cases of CLA.

## CONCLUSION

In conclusion, this study showed that it is technically feasible to produce 3D-VR reconstructions of patient-specific lung anatomy in children with CLA based on spirometry-controlled chest CECT images. A hybrid approach using VR and AI to analyse CT scan images led to an improved visualization of CLA lesions in relation to lung segment borders and local bronchovascular anatomy. Agreement in defining CLA extension based on lung segments increased between radiologist and surgeon after 3D-VR visualization. The paediatric thoracic surgeon changed the preferred surgical approach in the majority of cases after viewing the 3D-VR images in addition to conventional 2D-CT images. The successful representation of these images could pave the way for more advanced preoperative planning and a more accurate selection of suitable cases for lung-sparing intervention.

## Supplementary Material

ezad014_Supplementary_Data

## Data Availability

All relevant data are within the manuscript and its Supporting Information files.

## Notes

Presented at European Pediatric Surgery Association Congress 2022, Tel Aviv, Israel, 30 June 2022.

## ABBREVIATIONS

3D: Three-dimensional
AI: Artificial intelligence
BPS: Bronchopulmonary sequestration
CECT: Contrast-enhanced computed tomography
CLA: Congenital lung abnormality
CPAM: Congenital pulmonary airway malformation
CT: Computed tomography
LLL: Left lower lobe
RLL: Right lower lobe
VR: Virtual reality
