# Supplementary material for: Preoperative visualization of congenital lung abnormalities: hybridizing artificial intelligence and virtual reality
Source: Eur J Cardiothorac Surg. 2023 Jan 16;63(1):ezad014. doi: 10.1093/ejcts/ezad014 (PMC10481780; doi:10.1093/ejcts/ezad014)
Supplement: ezad014_Supplementary_Data [file ezad014_Supplementary_Data.zip › Supplementary material Revised.docx]

**Supplementary material**

**Supplementary Questionnaire A: Imaging of CLA with 2D-CT imaging & 3D VR imaging**

| **Question** | **Strongly disagree – strongly agree** |
| --- | --- |
| The size of the CLA lesion can be well assessed from this imaging modality. | 1 – 2 – 3 – 4 - 5 |
| The affected lung lobe(s) by CLA can be well assessed from this imaging modality. | 1 – 2 – 3 – 4 - 5 |
| The affected lung segment(s) by CLA can be well assessed from this imaging modality. | 1 – 2 – 3 – 4 - 5 |
| **Undermentioned statements are in case surgical indication arises** | |
| The pulmonary artery branches that need to be ligated can be determined from this imaging modality. | 1 – 2 – 3 – 4 - 5 |
| The pulmonary vein branches that need to be ligated can be determined from this imaging modality. | 1 – 2 – 3 – 4 - 5 |
| The bronchial branches that need to be ligated can be determined from this imaging modality. | 1 – 2 – 3 – 4 - 5 |
| Lung segments that potentially can be spared, can be determined from this imaging modality. | 1 – 2 – 3 – 4 - 5 |
| Surgical strategy ((multi)segmentectomy or lobectomy) can be decided upon based on this imaging modality. | 1 – 2 – 3 – 4 - 5 |
| This modality prepares me for surgery. | 1 – 2 – 3 – 4 - 5 |
| I am likely to consult this scan in the pre-operative planning. | 1 – 2 – 3 – 4 - 5 |
| I am likely to consult this scan during surgery. | 1 – 2 – 3 – 4 - 5 |

1. In which lung lobe(s) do you think the CLA lesion is located?

­­______________________________________________________

1. In which lung segment(s) do you think the CLA lesion is located?

______________________________________________________

1. Would you perform segmentectomy?

if Yes: which segments?

if No: lobectomy?

______________________________________________________

1. Comments on image modality:

______________________________________________________

**Supplementary Figure A: Image segmentation and quantitative assessment**

A+B+C) 2D views of patients’ CT scans, showing AI-based automatic segmentation of the segments of the right lower lobe and the bronchovascular anatomy of the right lung. D) 3D visualization of all segmented structures, including CPAM, of the right lower lobe. E) Quantitative assessment of the right lower lobe, showing the volumes, percentage of emphysema and lung density per segment.

*2D: Two-dimensional, 3D: Three-dimensional, CPAM: congenital pulmonary airway malformation, CT: Computed tomography*

**Supplementary Figure B: Right lower lobe segmentation: CPAM and scimitar vein of patient 1**

A) 3D reconstruction in 3D-Slicer of patient 1. Ventral view, showing CPAM localization in segments 7 and 8. Involvement of segment 10 cannot be determined in this view. B) PulmoVR view of all right lower lobe segments, including CPAM. C) PulmoVR view of venous drainage of the lung, including the scimitar vein that drains through the CPAM into the inferior vena cava (not visualized). D) Pulmo3D reconstruction, showing the CPAM and the scimitar vein in ventral view.

*3D: Three-dimensional, CPAM: congenital pulmonary airway malformation, S: segment*

**Supplementary Figure C: Left upper lobe segmentation: CPAM of patient 2**

A) Dorsal Pulmo3D view of left upper lobe. B) Lateral Pulmo3D view of left upper lobe, showing that all segments are affected.

*CPAM: congenital pulmonary airway malformation, S: segment.*

**Supplementary Video A:** Pulmo3D right lower lobe visualization of patient 3: BPS in blue shown in segment 6 (yellow) and 7 (orange). Other lung segments are not affected.

**Supplementary Video B:** *BPS in left lower lobe of patient 5*

PulmoVR left lower lobe visualization of patient 5: BPS in segment 10 (light blue) and segment 9 (mint green) with venous drainage into azygos vein and hemiazygos vein (blue). Segment X (white) is located frontal-medial.

**Supplementary Table A: Lung segment volumes per patient**

| Patient | R6 | R7 | R8 | R9 | R10 | RX | L1+2 | L3 | L4 | L5 | L6 | L7+8 | L9 | L10 | LX |
| --- | --- | --- | --- | --- | --- | --- | --- | --- | --- | --- | --- | --- | --- | --- | --- |
| #1 | 237.2 | 101.8 | 238.1 | 52.7 | 259.2 |  |  |  |  |  |  |  |  |  |  |
| #2 |  |  |  |  |  |  | 105.1 | 198.8 | 100.1 | 131.4 |  |  |  |  |  |
| #3 | 220.8 | 173.3 | 133.5 | 343.6 | 230.4 |  |  |  |  |  |  |  |  |  |  |
| #4 | 85.5 | 85.0 | 69.6 | 14.5 | 141.8 | 53.0 |  |  |  |  |  |  |  |  |  |
| #5 |  |  |  |  |  |  |  |  |  |  | 55.4 | 149.4 | 65.5 | 82.6 | 43.9 |

Lung volumes in milliliters(mm). RX is an additional lung segment that was found in the right lung. LX is an additional lung segment of the left lung. R or L corresponds to the side of the lung, and the number corresponds with the lung segment number.

**Supplementary Table B: Automated lobe-segment-lesion volume calculation**

| Patient # | Lung lobe (CT) | Total lobe volume (ml) | Affected lung segment(s) (VR) | Volume of affected segment(s) (ml) | Affected lung volume per lobe (%) | Potentially spared lung volume per lobe (%) |
| --- | --- | --- | --- | --- | --- | --- |
| 1 | RLL | 889 | Segment 7 + 8 + 10 | 599 | 67.4 | Basal segmentectomy: 27 |
| 2 | LUL | 535 | Segment 1-2 + 3 + 4 + 5 | 536 | 100 | Lobectomy: 0 |
| 3 | RLL | 1102 | Segment 6 + 7 | 394 | 35.7 | 64 |
| 4 | RLL | 449 | Segment 6 + 7 + 10 + X | 365 | 81.2 | Lobectomy: 0 |
| 5 | LLL | 396 | Segment 9 + 10 + X | 192 | 48.3 | 52 |

*AR: Augmented reality, CT: Computed tomography, LLL: Left lower lobe, LUL: Left upper lobe, ml: Milliliters, RLL: Right lower lobe, VR: Virtual reality*
