# Supplementary figures and images for: Preoperative visualization of congenital lung abnormalities: hybridizing artificial intelligence and virtual reality
Source: Eur J Cardiothorac Surg. 2023 Jan 16;63(1):ezad014. doi: 10.1093/ejcts/ezad014 (PMC10481780; doi:10.1093/ejcts/ezad014)

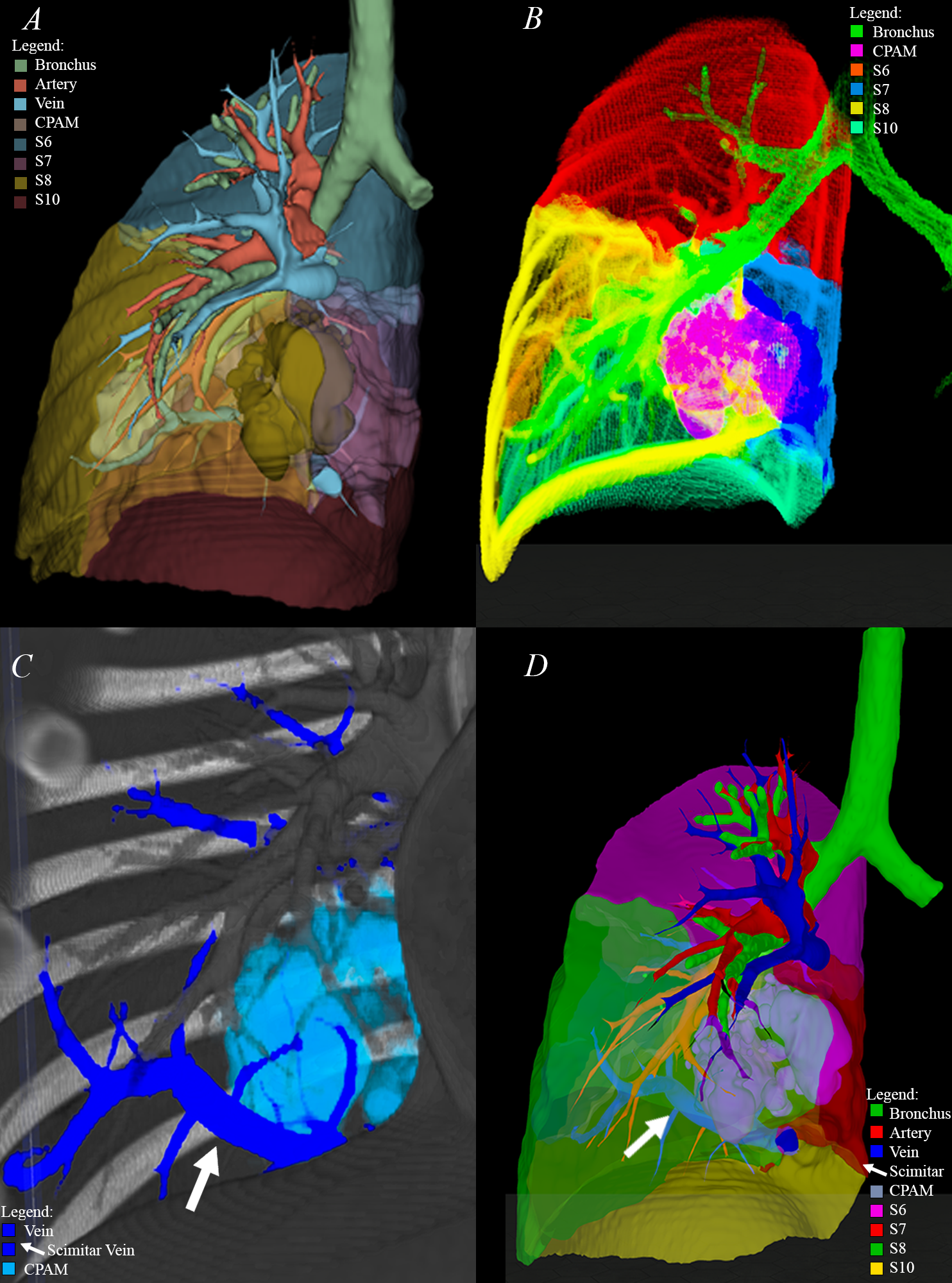

Supplement: ezad014_Supplementary_Data [file ezad014_Supplementary_Data.zip › SupplementaryFigureB.png]

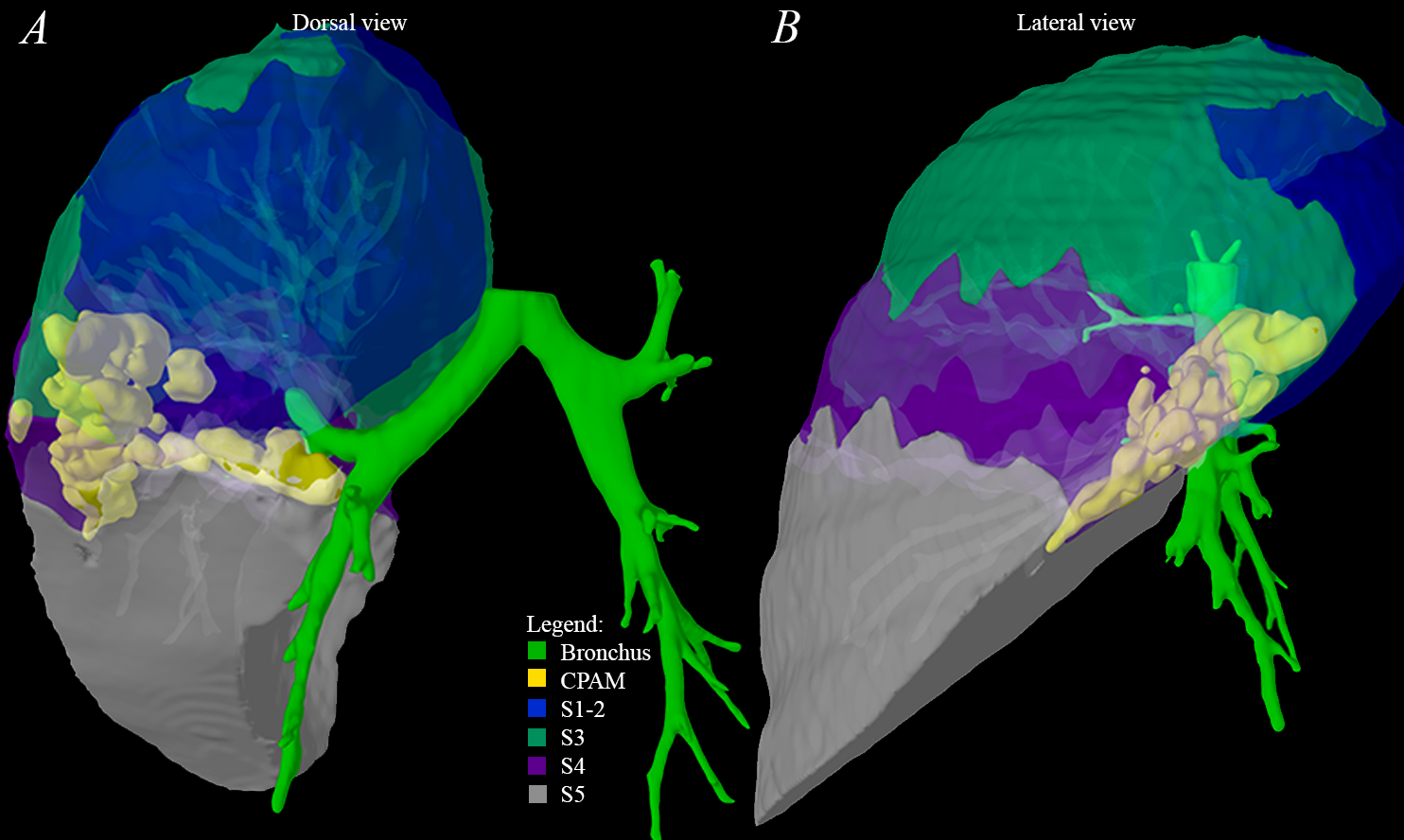

Supplement: ezad014_Supplementary_Data [file ezad014_Supplementary_Data.zip › SupplementaryFigureC.png]

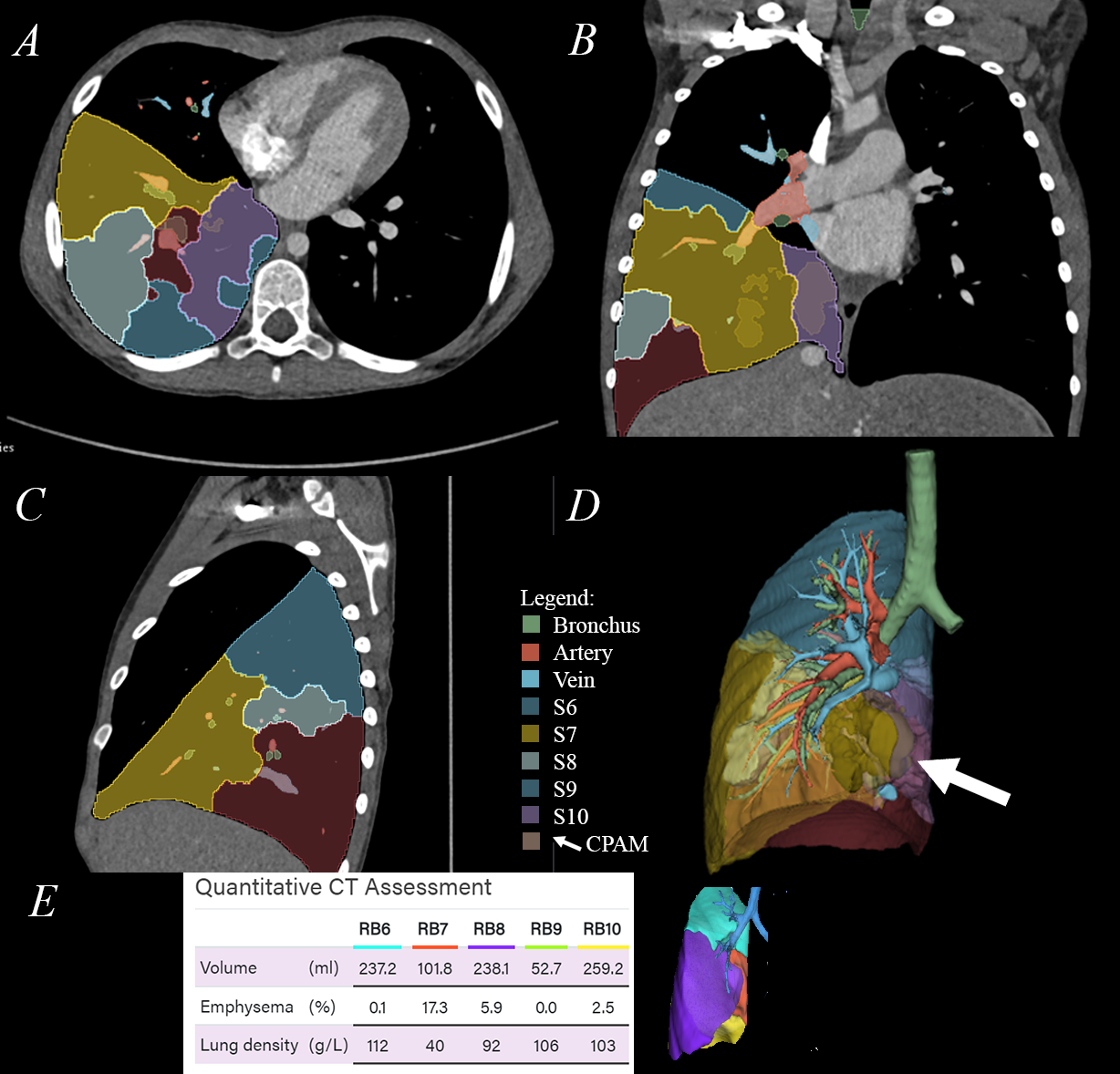

Supplement: ezad014_Supplementary_Data [file ezad014_Supplementary_Data.zip › SupplementaryFigureA.png]
